# Fourth Annual Conference of the Canadian Hospital Association in Montreal

**Published:** 1910-04-16

**Authors:** 


### FOURTH ANNUAL CONFERENCE OF THE CANADIAN HOSPITAL ASSOCIATION IN MONTREAL.

On Monday, March 28, the annual conference of the Canadian Hospital Association opened in the Royal Victoria Hospital, Montreal. Mr. H. E. Webster, superintendent of the Victoria, presided over a fairly large assembly, the greater part of whom were ladies. The Mayor of Montreal, who is also a well-known medical man, Dr. Guerin, was present, and made an apt speech, in which he warmly welcomed the hospital delegates to Montreal. Mr. Webster made a commendably brief opening speech in view of the fact that the programme was long.

### Hospital Accounting.

The first paper, on "The relation of the Training School to the Hospital Deficit Problem," was read by Dr. Edith Beatty, of Toronto, for Miss Charlotte Aikens, Detroit.

Dr. R. W. Bruce Smith, inspector of hospitals, Ontario, read an extremely instructive paper on "Hospital Accounting," in which it was shown how a careful system of supervising expenditure in hospitals resulted in great saving. The method employed with great success in the General Hospital, Toronto, was described by the speaker, who contended that there should be a uniform system of accounting in hospitals. Dr. J. U. E. Browne, superintendent of Toronto General Hospital, who is responsible for the introduction of the present method of accounting in that institution, more fully explained the method, and said that it had been the means of saving thousands of dollars.

A demonstration was then given of a room in a private residence arranged for conducting an operation.

Dr. Helen MacMurchy, Toronto, read a paper at the afternoon session on the subject of "Social Service." By this was meant to assist those who left the hospital to secure positions when they were unable to return to their former employment, and also to render help to those who were in need of other than medical aid. Work in this direction was first begun in the Johns Hopkins Hospital, Baltimore, but has been more fully developed in the Massachusetts General Hospital.

Dr. W. J. Dobbie, superintendent, Toronto Hospital for Consumptives, exhibited certain simple devices which had been found useful.

### Noise.

There was then read a paper which interested your correspondent intensely, as it is a matter on which he holds decided views. The title of the paper was "Noise," and, naturally, referred to noise in connection with hospitals. Too little attention is paid to this matter, which is one that affects most intimately the well-being of patients. With a certain class of patients quiet is essential to recovery, and in these days when neurasthenia and nervous complaints are rampant a quiet hospital would seem to be a necessity. But how many hospitals are situated in a neighbourhood free from noise, or even comparatively free from noise? With the advent of locomotion by means of electricity and of the motor, life in a large city is more nerve-jarring than ever before, and it therefore behoves those responsible for the situation of a hospital to insist if possible that it be removed far from the madding crowd and away from the noise of city traffic. Further, the internal arrangements of hospitals should be such that noise inside is reduced to a minimum. This is by no means always the case, and the medical and nursing staffs are not infrequently sinners in this respect. There would appear to be no valid reason why walls of hospitals should not be so constructed as to deaden sound, in the way that walls of conservatoires of music are constructed. At any rate, every effort should be put forth to render hospitals havens of peace as well as body-patching institutions. There is no doubt that the aspect of the subject discussed by Dr. Boyce has not received the consideration which its importance deserves.

### The Evening Sitting.

After the reading of this paper the members of the Conference paid a visit to the Montreal General Hospital, which is in process of reconstruction.

The evening sitting of the Conference was wholly devoted to hospital construction. Mr. J. H. S. Parke, the superintendent of the Montreal General Hospital, explained the plans for the reconstruction of the institution.

Afterwards, in one of the operating theatres of the Victoria Hospital, Mr. Meyer J. Sturm, of Chicago, hospital architect, gave an address on "Practical Idealism in Planning Hospitals." Mr. Sturm illustrated his address by showing on the screen plans of various hospitals.

### Hospital Construction.

On Tuesday, March 29, of the papers read in the morning one was of outstanding merit. This was read by Dr. C. K. Holmes, of Cincinnati, Ohio. The subject-matter of the paper was excellent, for Dr. Holmes is an enthusiast in hospital construction, and is, moreover, possessed of practical ideas, guided by highly trained knowledge. There is at the present time on this continent a spirit of emulation as regards hospital construction. Three large new hospitals are in course of construction or about to be built in different parts of the United States and Canada. These are the General Hospital, Toronto, an immense undertaking, the buildings of which will more or less cover an area of between nine and ten acres; the Cincinnati General Hospital, also intended to accommodate a large number of patients; and the Peter Bergham, Baltimore, which is also said to be the last word in hospital construction. The initiators of these three hospitals have travelled almost all over the civilised world in order to gain the latest views on hospital construction. Dr. Holmes, in particular, has traversed Europe, Great Britain, and America in his quest for new ideas, and has absolutely closely inspected every hospital of note in the world. In his paper read in Montreal Dr. Holmes discussed, praised, and criticised features of these many institutions, illustrating his words by excellent views on the screen. He was particularly eloquent on the ventilating question, condemning in most emphatic manner the plenum system of ventilation and declaring himself a whole-souled advocate of the upward draught method. He stated that he was of the opinion that 24 was the number best adapted to each ward of a large hospital, and that heating by hot water was the most effective and healthy mode of heating. In the new Cincinnati hospital the roofs will be flat, and used as roof gardens for the benefit of patients.

Dr. J. U. E. Browne gave a careful and exhaustive description, illustrated on the screen, of the buildings of the future Toronto General Hospital. The entire meeting was a great success, both from a technical and social point of view.

